# Lateral transfer of mRNA and protein by migrasomes modifies the recipient cells

**DOI:** 10.1038/s41422-020-00415-3

**Published:** 2020-09-29

**Authors:** Mingli Zhu, Qin Zou, Rongyao Huang, Ying Li, Xudong Xing, Jianhuo Fang, Liang Ma, Lifei Li, Xuerui Yang, Li Yu

**Affiliations:** 1 State Key Laboratory of Membrane Biology, Tsinghua University-Peking University Joint Center for Life Sciences, Beijing Frontier Research Center for Biological Structure, School of Life Science, Tsinghua University, Beijing, 100084 China; 2 Joint Graduate Program of Peking-Tsinghua-National Institute of Biological Science, Tsinghua University, Beijing, 100084 China; 3 MOE Key Laboratory of Bioinformatics, Tsinghua University, Center for Synthetic & Systems Biology, Tsinghua University, School of Life Science, Beijing, 100084 China

**Keywords:** Extracellular signalling molecules, Organelles

Dear Editor,

Migrasomes are recently discovered vesicular organelles which form on the retraction fibers (RFs) of migrating cells.^1^ Once detached from cells, migrasomes can rupture and release their luminal contents^1^ in a process named migracytosis. Recently, migrasomes have been shown to play an important role in zebrafish organ morphogenesis by releasing chemokine signals to defined regions of the embryo.^2^ Thus, migracytosis is considered as a major mechanism for migrasomes to carry out their functions. It has been frequently observed that intact migrasomes can be engulfed by surrounding cells,^1^ and this has been proposed as a potential mechanism for lateral transfer of cellular contents between cells. However, it is not clear whether the lateral transfer of cellular contents by migrasomes has functional consequences for the recipient cells.

We found that migrasomes can be stained by SYTO 14 (Fig. 1a), a dye which emits a fluorescent signal only after binding to nucleic acids.^3^ To determine the nature of the nucleic acids in migrasomes, we fixed and permeabilized cells, stained the cells with SYTO 14 and then treated them with DNase or RNase. We found that only RNase treatment reduced the SYTO 14 signal inside the migrasomes (Fig. 1c, d; Supplementary information, Fig. S1b), and, adding an RNase inhibitor protected the SYTO 14 signal in RNase-treated migrasomes (Supplementary information, Fig. S1c, d). This indicates that RNAs are present in migrasomes. On average, 30% of migrasomes contain RNA (Fig. 1b), and the intensity of SYTO 14 staining varies considerably between migrasomes (Supplementary information, Fig. S1a).

**Fig. 1:**
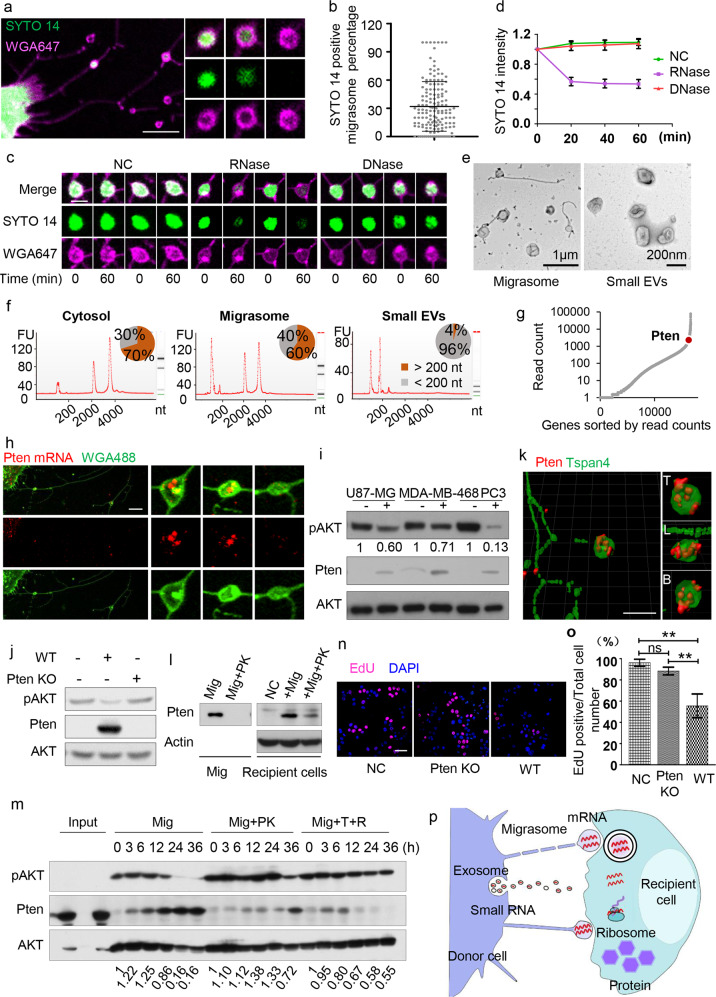
Lateral transfer of mRNA and protein by migrasomes modifies the recipient cells. **a** Representative image of an L929 cell stained with SYTO 14 and WGA 647. Scale bar, 10 μm. **b** The percentage of SYTO 14-positive migrasomes was quantified in L929 cells (*n* = 150 cells from three independent experiments). Data are presented as means ± SD. **c** Representative images of SYTO 14-positive migrasomes treated with PBS (negative control, NC), 10 µg/mL RNase, or 30 u/mL RNase-free DNase for 60 min. Scale bar, 2 μm. **d** Quantification of SYTO 14 mean intensity in migrasomes before and after enzyme treatment (*n* = 90 migrasomes from three independent experiments). Data are presented as means ± SD. For NC and DNase treatment, *P* = 0.5080; for NC and RNase treatment, *P* < 0.0001. **e** TEM images of purified migrasomes and small EVs. **f** Electropherograms of total RNA isolated from the cytosol, migrasome and small EV fractions of mouse L929 cells. Size distributions of the RNAs are shown on the electropherogram traces (red lines), and the percentages of long (> 200 nt) and short (< 200 nt) RNA species are shown as pie charts. **g** All genes with at least one read in the migrasome RNA-seq dataset are sorted, from left to right, by their read counts (shown on the Y-axis). *Pten* is highlighted on the dot plot. **h** Detection of *Pten* mRNA localization in migrasomes. Scale bar, 5 μm. **i** Purified migrasomes (8 μg) were incubated with U87-MG, MDA-MB-468, and PC3 cells for 24 h, respectively. Protein levels were assessed by western blotting. **j** Migrasomes (8 μg) were purified from WT or *Pten* KO L929 cells and incubated with MDA-MB-468 cells for 24 h. pAKT and Pten levels were analyzed by western blotting. **k** Representative images of WT L929 cells stained with antibody against Pten and imaged by SIM microscopy. Scale bar, 5 μm. **l** Left panel: Purified migrasomes (10 μg) were treated without (Mig) or with (Mig+PK) 50 μg/mL Proteinase K for 30 min, then analyzed by western blotting for the presence of Pten. Right panel: Purified migrasomes (10 μg) treated with or without 50 μg/mL Proteinase K for 30 min were incubated with MDA-MB-468 cells for 24 h. Cellular Pten levels were assessed by western blotting. NC, negative control (untreated cells). **m** Purified migrasomes (20 μg) were treated with 50 μg/mL Proteinase K (Mig+PK) or 10 μg/mL RNase plus 0.1% Triton X-100 (Mig+T+R), and incubated with MDA-MB-468 cells for 3, 6, 12, 24, and 36 h. Protein levels were assessed by western blotting. **n** Representative images of EdU incorporation in MDA-MB-468 cells treated for 18 h with PBS (NC), migrasomes (8 μg) isolated from wild-type cells (WT) or migrasomes (8 μg) isolated from *Pten* KO cells (Pten KO). **o** The percentage of EdU-positive cells was measured and normalized to control cells (*n* = 3 independent experiments). Data are presented as means ± SD. **p** Model of mRNA transfer by migrasomes.

To characterize the RNA species in migrasomes, we isolated migrasomes from L929 cells. Migrasomes adhere to the bottom of culture dishes, while other known extracellular vesicles (EVs) are present in the medium. Therefore, to ensure the purity of migrasomes, we disposed of the culture medium and washed the dishes before purifying migrasomes. In theory, this should remove the majority of other known EVs and reduce the potential contamination. A fraction of purified migrasome are attached to retraction fibers and contain luminal vesicles (Fig. 1e; Supplementary information, Fig. S2a, b), and thus purified migrasomes can be distinguished morphologically from other known EVs. Biochemical analysis showed that migrasomes are enriched with Itga5 and contain actin (Supplementary information, Figs. S2c and S9e), as described before.^1,4^ No ER or mitochondrial markers are found in purified migrasomes (Supplementary information, Figs. S2c and S9e). Moreover, migrasomes contain the tetraspanin CD63, but not Alix or Tsg101 (Supplementary information, Figs. S2c and S9e), which rules out contamination with small EVs (Supplementary information, Fig. S2d).

To rule out possible contamination with microvesicles (MVs), which are also derived from the plasma membrane, we carried out 4D imaging of L929 cells. We did not observe direct budding of MVs from the plasma membrane (Supplementary information, Fig. S3a and Movie S1). In contrast, we observed extensive migrasome formation (Supplementary information, Fig. S3a and Movie S1), which suggests that the majority of plasma membrane-derived vesicles generated by L929 cells are migrasomes. The sizes of “MVs” isolated from medium range from 100 nm to 1 µm, which is similar to the case of migrasomes detected by negative staining in situ (Supplementary information, Fig. S3b, c). Morphologically, “MVs” from culture medium of L929 cells contain intraluminal vesicles, which is the characteristic feature of migrasomes (Supplementary information, Fig. S3d). Moreover, the amount of “MVs” isolated from the culture medium was significantly reduced after blocking migrasome formation (Supplementary information, Fig. S4a–c), and significantly increased after inducing migrasome formation (Supplementary information, Fig. S4d–f), which implies that the “MVs” in the culture medium are detached migrasomes. Consistent with this notion, immunostaining showed that Annexin A1, a well-known marker for MVs, is highly enriched in migrasomes (Supplementary information, Fig. S5a, b), and the purified migrasomes and “MVs” are enriched with the same set of protein markers (Supplementary information, Fig. S5c). Put together, these data argue that at least in our cell culture conditions, “MVs” are detached migrasomes.

As the control, small EVs were isolated from the same cells. Transmission electron microscopy and western blotting confirmed clean isolation of the migrasomes and small EVs (Fig. 1e; Supplementary information, Figs. S2c, d and S9e, f). Total RNA was then extracted from the migrasomes and small EVs. The overall length distribution of the RNA from migrasomes and small EVs is very different (Fig. 1f). In migrasomes, most of the RNA species are long (> 200 nt), while in small EVs, the length distribution is dominated by small RNA species (Fig. 1f).

Total RNA sequencing (total RNA-seq) revealed that migrasome RNA (after depletion of ribosomal RNA) is mainly composed of mRNA species (Supplementary information, Fig. S6a). The abundance of each RNA species in the migrasome or cytosol fraction was quantified by RNA-seq read counts in duplicated experiments (Supplementary information, Fig. S6b and Table S1). Differential RNA expression analysis then revealed a group of mRNA species that are enriched in migrasomes (Supplementary information, Fig. S6c). These over-represented mRNAs are highly enriched in cellular processes related to metabolism, intracellular transportation, cell junctions, vesicle fusion, assembly of subcellular and membrane structures, etc (Supplementary information, Fig. S6d).

We asked whether migrasomal mRNA can be transferred into recipient cells and then translated. We chose *Pten* as an example, as it was among the most abundant group of mRNAs in migrasomes (Fig. 1g; Supplementary information, Fig. S7a). The RNA-seq reads covered the entire *Pten* transcript (Supplementary information, Fig. S7b), which suggests that the full-length *Pten* mRNA is present in migrasomes. 5′-UTR of *Pten* mRNA can be detected in migrasomes (Supplementary information, Fig. S7c), suggesting that migrasomes contain full-length *Pten* mRNA. It is worth noting that we could not detect full-length *Pten* mRNA in small EVs (Supplementary information, Fig. S7c). In addition, single-molecule FISH and RT-PCR analysis of *Pten* also supported the presence of *Pten* mRNA in migrasomes (Fig. 1h; Supplementary information, Fig. S7d and Table S2).

To test the role of migrasomes in recipient cells, we added purified migrasomes from L929 cells into U87-MG, MDA-MD-468, and PC3 cells, none of which express Pten protein due to frame-shift mutations. We found that Pten protein was detected inside these cells (Fig. 1i; Supplementary information, Fig. S9a). Strikingly, in these cells, the pAKT signal is dramatically reduced (Fig. 1i; Supplementary information, Fig. S9a). This implies that adding migrasomes can cause accumulation of Pten protein and reduction of pAKT activity in the recipient cells. To confirm that the pAKT activity reduction was caused by Pten, we added migrasomes from wild-type (WT) or *Pten* knockout (KO) L929 cells (Supplementary information, Fig. S8a) into MDA-MB-468 cells. We found that Pten protein was detected in recipient cells incubated with migrasomes from WT cells, but not with migrasomes from *Pten* KO cells (Fig. 1j; Supplementary information, Fig. S9b). Moreover, pAKT levels are visibly reduced in cells treated with migrasomes from WT cells (Fig. 1j; Supplementary information, Fig. S9b).

Further characterization showed that migrasomes have a dose-dependent effect on the level of Pten protein and the reduction in the pAKT signal in recipient cells (Supplementary information, Figs. S8b and S9g). Moreover, the amount of Pten in recipient cells keeps rising and the pAKT signal keeps decreasing (Supplementary information, Figs. S8c and S9h), which suggests that new Pten protein is synthesized in recipient cells.

Pten protein is detected in migrasomes (Fig. 1k; Supplementary information, Fig. S2c). To distinguish the effect of migrasomal Pten protein vs *Pten* mRNA, we treated isolated migrasomes with proteinase K. We found that Pten protein was completely removed from migrasomes (Fig. 1l; Supplementary information, Fig. S9c) while the migrasomal morphology remained intact (Supplementary information, Fig. S8d). Although Pten protein was completely removed from the purified migrasomes by proteinase K, Pten protein was still detected in the recipient cells (Fig. 1l; Supplementary information, Fig. S9c). This confirms that *Pten* mRNA is transferred into recipient cells and then translated.

Although this observation is clear and can be reliably repeated, the fact that proteinase K can degrade Pten protein in migrasomes still surprised us, as the migrasome membrane should protect Pten protein from proteinase K-mediated degradation. This observation raised the possibility that the membrane of purified migrasomes is leaky. To test this, we added the water-soluble dye Cy-5 and 40 kD dextrans into purified migrasomes. We found that both Cy-5 and dextrans can enter migrasomes (Supplementary information, Fig. S8d), which suggests that purified migrasomes are indeed leaky.

We treated migrasomes with proteinase K to remove Pten protein, or Triton X-100 plus RNase A to remove *Pten* mRNA. In recipient cells incubated with proteinase K-treated migrasomes, very little Pten protein was detected 12 h after adding the migrasomes; however, at 36 h after adding the migrasomes, Pten protein was clearly detected, and the pAKT signal was markedly reduced (Fig. 1m; Supplementary information, Fig. S9d). In cells incubated with migrasomes treated with Triton X-100 plus RNase A, Pten protein was detected at 6 h after migrasome addition (Fig. 1m; Supplementary information, Fig. S9d). However, 36 h after adding migrasomes, there was very little Pten protein signal left (Fig. 1m; Supplementary information, Fig. S9d). Thus, both migrasomal *Pten* mRNA and protein contribute to modulation of the pAKT level in the recipient cell, with Pten protein modulating the pAKT level at earlier time points and *Pten* mRNA playing a more important role at later time points.

Finally, we investigated whether or not the laterally transferred *Pten* mRNA and Pten protein has any functional consequence in the recipient cells. It is well known that Pten upregulation can inhibit cancer cell proliferation.^5,6^ To test whether migrasome-mediated transfer of *Pten* mRNA and Pten protein can inhibit the proliferation of Pten-deficient cells, we added migrasomes isolated from wild-type and *Pten* KO cells into MDA-MB-468 cells. We found that the proliferation of MDA-MB-468 cells was inhibited more strongly by migrasomes from wild-type cells than by migrasomes from *Pten* KO cells (Fig. 1n, o). Thus, lateral transfer of *Pten* mRNA and Pten protein by migrasomes inhibits the proliferation of the recipient cells.

In summary, our study reveals that migrasomes contain mRNAs and proteins, which can be laterally transferred into recipient cells. The mRNAs are then translated into proteins which can functionally modify the recipient cell (Fig. 1p). We speculate that the lateral transfer of mRNA and protein may emerge as an important mechanism by which migrasomes carry out their physiological functions. The RNA sorting and transport mechanisms remain to be identified.

## Supplementary information

Supplementary figures and methods

Supplementary information, Table S1

Supplementary information, Table S2

Supplementary information, movie S1

Supplementary information, movie S1 legend
