# Supplementary material for: Lateral transfer of mRNA and protein by migrasomes modifies the recipient cells
Source: Cell Res. 2020 Sep 29;31(2):237–40. doi: 10.1038/s41422-020-00415-3 (PMC8026638; doi:10.1038/s41422-020-00415-3)
Supplement: Supplementary file 1 — Supplementary figures and methods [file 41422_2020_415_MOESM1_ESM.pdf]

Figure S1

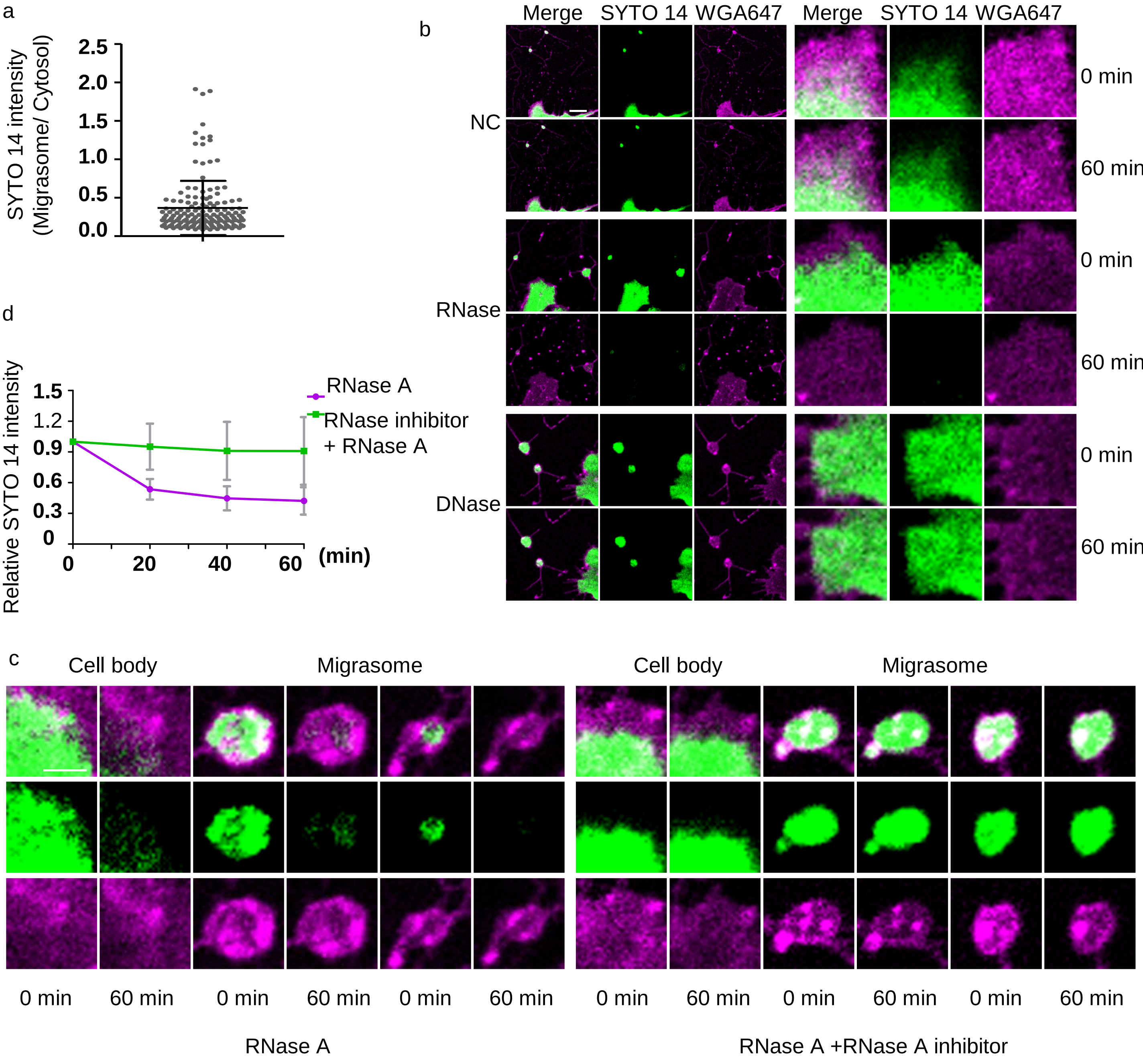

a
SYTO 14 intensity (Migrasome/ Cytosol)
2.5
2.0
1.5
1.0
0.5
0.0
b
Merge
SYTO 14
WGA647
Merge
SYTO 14
WGA647
NC
RNase
DNase
0 min
60 min
0 min
60 min
0 min
60 min
d
Relative SYTO 14 intensity
1.5
1.2
0.9
0.6
0.3
0
0
20
40
60
(min)
RNase A
RNase inhibitor + RNase A
c
Cell body
Migrasome
Cell body
Migrasome
0 min
60 min
0 min
60 min
0 min
60 min
0 min
60 min
0 min
60 min
0 min
60 min
RNase A
RNase A +RNase A inhibitor

Supplementary information, Fig. S1. SYTO 14 stains RNA in migrasomes.

(a) The mean intensity of SYTO 14 was measured and compared in migrasomes and cytosol. ROIs of equal size were randomly selected for analysis from migrasomes and cytosol. n=150 cells from three independent experiments. Data are presented as mean±SD.

(b) Representative images of L929 cells treated with PBS (NC), RNase, and DNase for 0 and 60 min. Left: both migrasomes and cytosol are shown. Right: enlarged images of the cytosol. Enlarged migrasomes from Fig. S1b are shown as in Fig. 1c. Green signal, SYTO 14. Scale bar, 5 μm.

(c) Representative image of L929 cells stained with SYTO 14 and WGA488 and treated with 10 μg/ml RNase A, or 4 u/μl RiboLock RNase inhibitor plus 10 μg/ml RNase A. Left panel: enlarged images of cell bodies and migrasomes treated with RNase A. Right panel: enlarged images of cell bodies and migrasomes treated with RNase inhibitor plus RNase A. Scale bar, 2 μm.

(d) Quantification of SYTO 14 intensity in migrasomes before and after treatment with 10 μg/ml RNase A, or 4 u/μl RiboLock RNase inhibitor plus 10 μg/ml RNase A. n=60 migrasomes from three independent experiments. Data are presented as mean±SD.

Figure S2

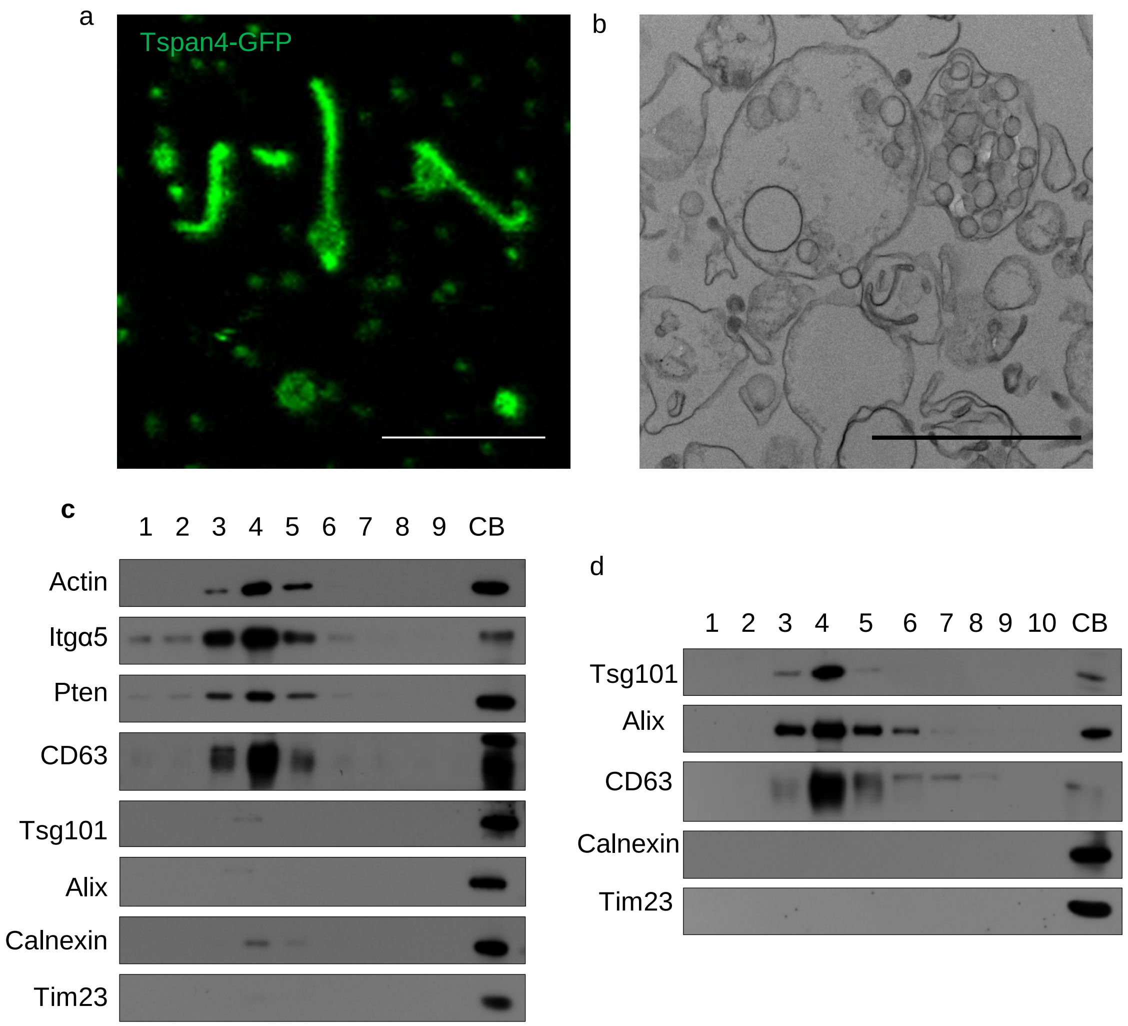
a
Tspan4-GFP
b
c
1 2 3 4 5 6 7 8 9 CB
Actin
Itgα5
Pten
CD63
Tsg101
Alix
Calnexin
Tim23
d
1 2 3 4 5 6 7 8 9 10 CB
Tsg101
Alix
CD63
Calnexin
Tim23

Supplementary information, Fig. S2. Characterization of purified migrasomes.
(a) Migrasomes from Tspan4-GFP-expressing L929 cells were purified and then observed by confocal microscopy. Fragments of retraction fibers are visible. Scale bar, 5 μm.
(b) Representative EM image of purified migrasomes after section preparation. Luminal vesicles are visible. Scale bar, 1 μm.
(c) Crude migrasomes were centrifuged in an OptiPrep density gradient. The different fractions (1-9) were analyzed by western blotting to determine the distribution of the cytosol marker Actin, the migrasome-enriched protein Itga5, the ER marker Calnexin, the small EV markers CD63, Tsg101 and Alix, and the mitochondrion marker Tim23. CB, cell bodies.
(d) Small EVs collected from the cell culture medium were centrifuged in a sucrose density gradient. The distribution of the small EV markers CD63, Alix and Tsg101, the ER marker Calnexin, and the mitochondrion marker Tim23 in the fractions was evaluated by western blotting. CB, cell bodies.

Figure S3

a

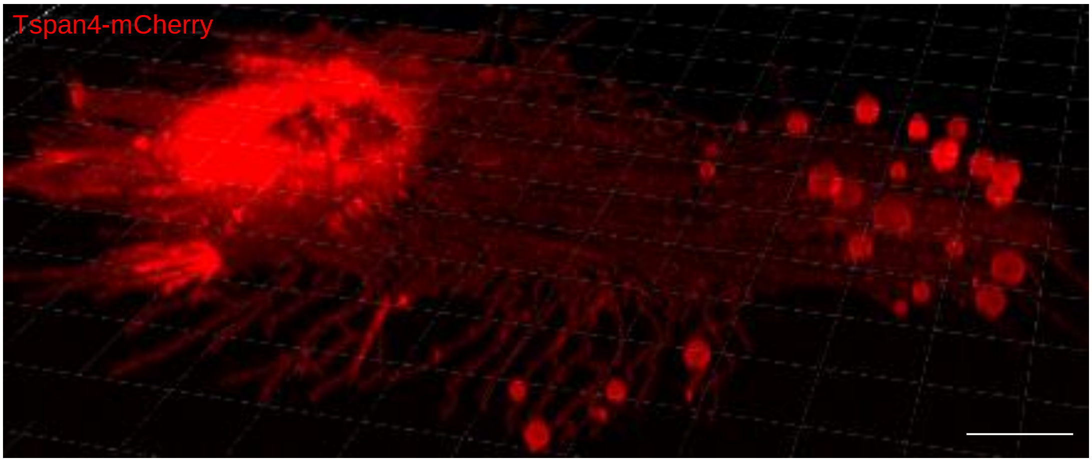

b

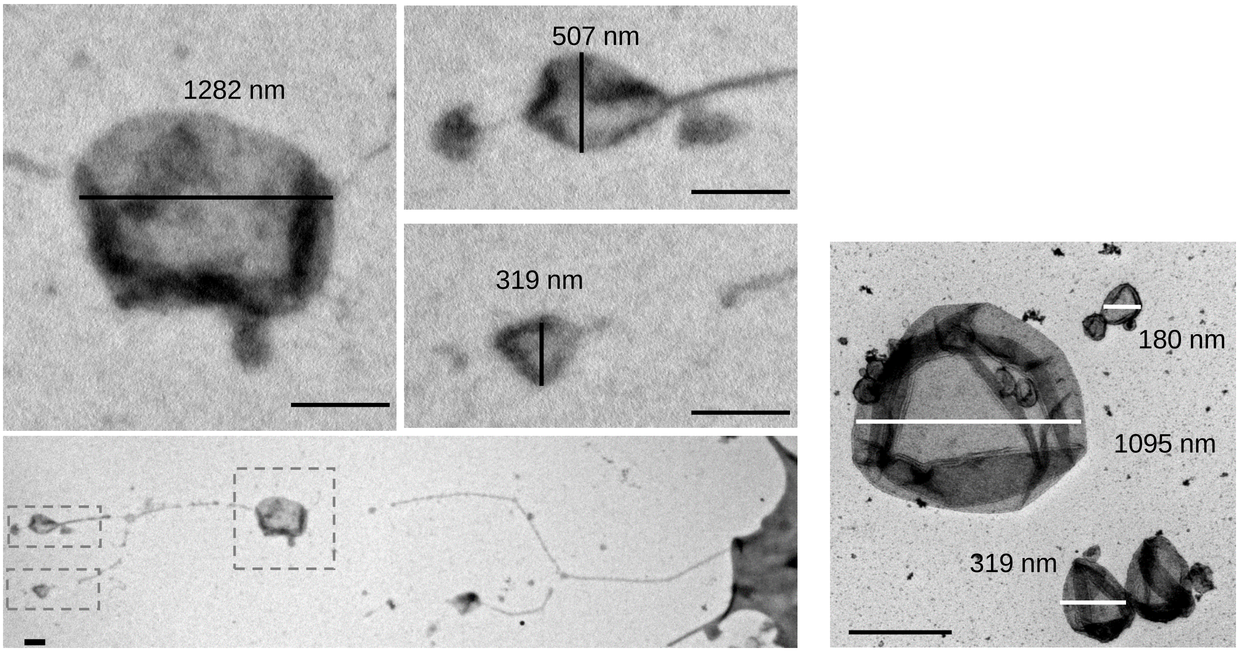

Migrasome in situ

Purified “MVs”

c

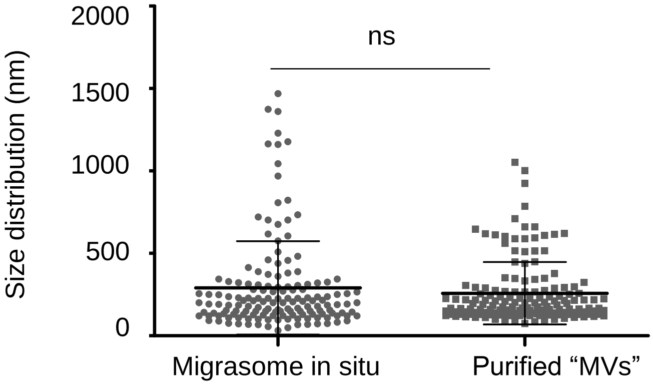

d

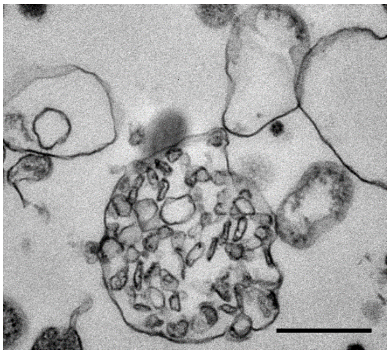

Supplementary information, Fig. S3. Purified “MVs” are morphologically similar to migrasomes.

(a) A representative image (from a 4-dimensional movie) of an L929 cell expressing the migrasome marker Tspan4-mCherry, Scale bar, 10 μm.

(b) Representative TEM image of migrasome generated by L929 cells in situ and purified “MVs”. L929 cells were cultured on grid and then subjected to negative staining. Scale bar, 500nm.

(c) Size distribution of migrasome generated by L929 cells in situ and purified “MVs”. N= 150 vesicles from three independent experiments. Data are presented as mean ± SD.

(d) Representative TEM images of ultra-thin section of purified “MVs”. Scale bar, 500nm.

Figure S4

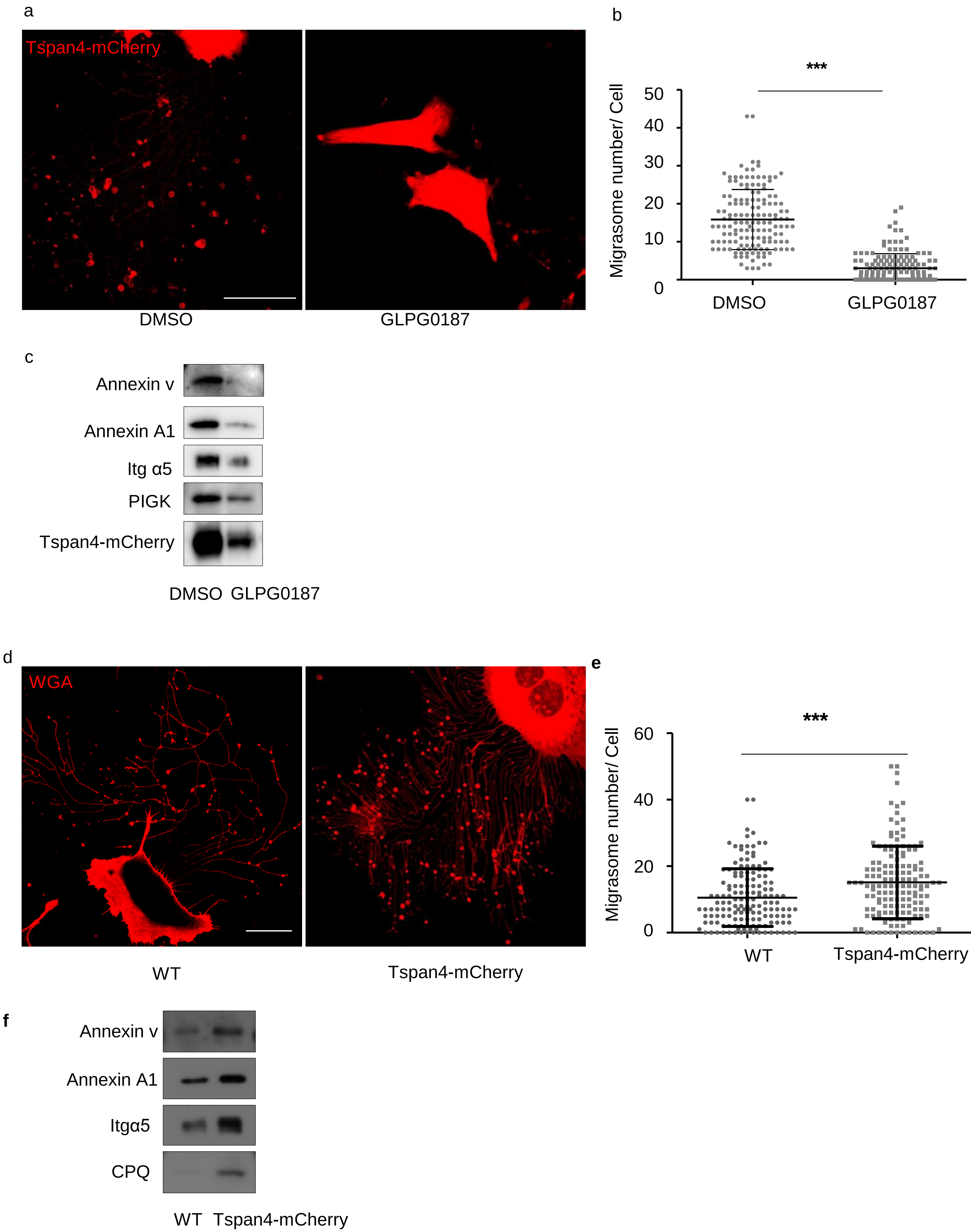
a
Tspan4-mCherry
DMSO
GLPG0187
b
***
Migrasome number/ Cell
50
40
30
20
10
0
DMSO
GLPG0187
c
Annexin v
Annexin A1
Itg α5
PIGK
Tspan4-mCherry
DMSO GLPG0187
d
WGA
WT
Tspan4-mCherry
e
***
Migrasome number/ Cell
60
40
20
0
WT
Tspan4-mCherry
f
Annexin v
Annexin A1
Itgα5
CPQ
WT Tspan4-mCherry

Supplementary information, Fig. S4. Blocking migrasome formation reduce purified “MVs” number and promoting migrasome formation increases the purified “MVs” number.

(a) Tspan4-mCherry L929 cells were seeded into 10 μg/ml fibronectin-coated dishes for 5 hours, then treated without or with 2.5 μM GLPG0187 for 8 hours. Representative confocal images are shown. Scale bar, 10 μm.

(b) Cells were treated without or with 2.5 μM GLPG0187 and the number of migrasomes per cell was counted. n=150 cells from three independent experiments. Data represent mean ± SD.

(c) Equal volumes of “MVs” were collected from the culture medium of control cells and GLPG0187-treated cells, then analyzed by western blotting with antibodies against the MV markers Annexin v and Annexin A1, and the migrasome-enriched proteins Integrin α5, PIGK, and Tspan4-mCherry.

(d) Equal numbers of WT or Tspan4-mCherry L929 cells were seeded into 10 μg/ml fibronectin-coated dishes for 12 hours, then fixed and imaged. Representative confocal images are shown. Scale bar, 20 μm.

(e) WT or Tspan4-mCherry L929 cells were imaged as in (a) and the number of migrasomes per cell was counted. n=150 cells from three independent experiments. Data represent mean ± SD.

(f) Equal volumes of “MVs” were collected from the culture medium of WT and Tspan4-mCherry L929 cells, then analyzed by western blotting with antibodies against the MV markers Annexin V and Annexin A1, and the migrasome-enriched proteins Integrin α5 and CPQ.

Figure S5

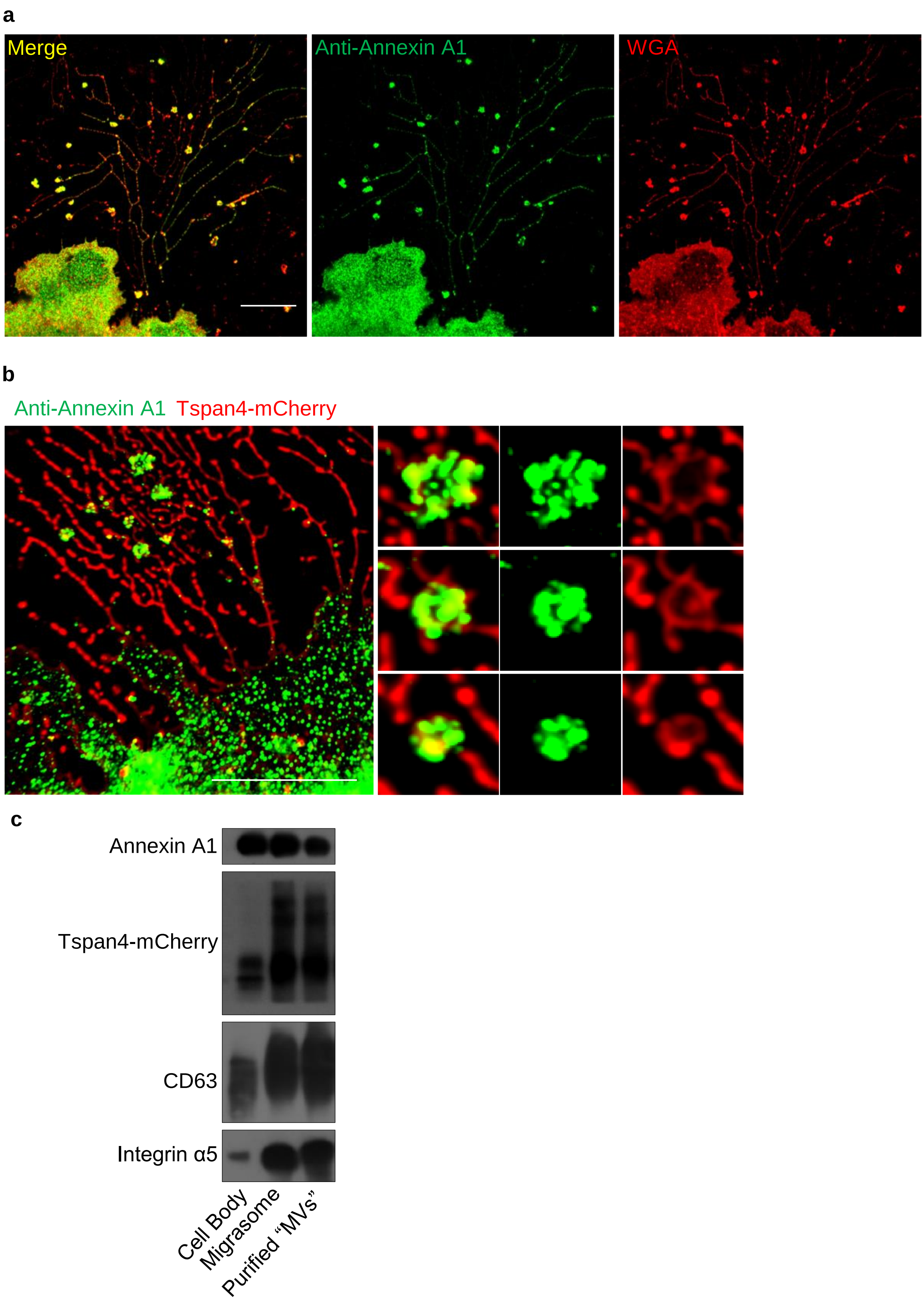
a
Merge
Anti-Annexin A1
WGA
b
Anti-Annexin A1 Tspan4-mCherry
c
Annexin A1
Tspan4-mCherry
CD63
Integrin α5
Cell Body
Migrasome
Purified "MVs"

Supplementary information, Fig. S5. Migrasomes and “microvesicles” have a similar biochemical signature.
(a) A representative confocal microscope image of an L929 cell stained with Annexin A1. Migrasomes are labeled by WGA. Scale bar, 10 μm.
(b) A representative SIM microscope image of an L929 cell stained with Annexin A1. Migrasomes are labeled by Tspan4-mCherry. Scale bar, 10 μm.
(c) 5 μg of cell bodies, migrasomes and MVs were analyzed by western blotting with antibodies against Annexin A1, CD63, Tspan4-mCherry and Integrin α5.

Figure S6

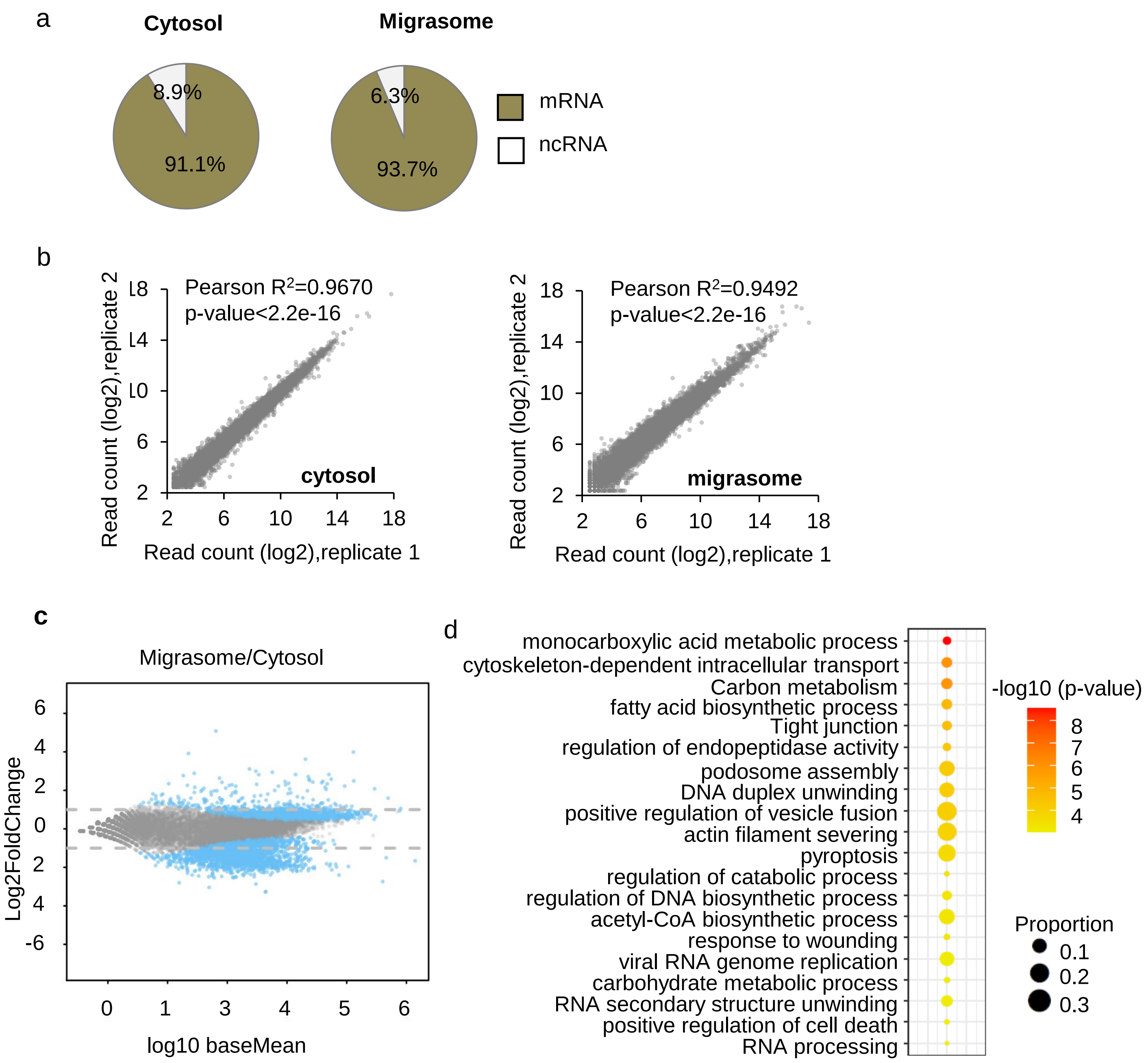
a
Cytosol
Migrasome
8.9%
91.1%
6.3%
93.7%
mRNA
ncRNA
b
Pearson R2=0.9670
p-value<2.2e-16
cytosol
Read count (log2),replicate 2
Read count (log2),replicate 1
Pearson R2=0.9492
p-value<2.2e-16
migrasome
c
Migrasome/Cytosol
Log2FoldChange
log10 baseMean
d
monocarboxylic acid metabolic process
cytoskeleton-dependent intracellular transport
Carbon metabolism
fatty acid biosynthetic process
Tight junction
regulation of endopeptidase activity
podosome assembly
DNA duplex unwinding
positive regulation of vesicle fusion
actin filament severing
pyroptosis
regulation of catabolic process
regulation of DNA biosynthetic process
acetyl-CoA biosynthetic process
response to wounding
viral RNA genome replication
carbohydrate metabolic process
RNA secondary structure unwinding
positive regulation of cell death
RNA processing
-log10 (p-value)
Proportion
0.1
0.2
0.3

Supplementary information, Fig. S6. Characterization of migrasomal RNA.
(a) Percentage compositions of mRNA and ncRNA in total RNA from migrasomes and cytosol of mouse L929 cells. Profiling was carried out by total RNA-seq.
(b) Left: Comparison of the L929 cytosol RNA-seq read counts in two biological replicates. Right: Comparison of the L929 migrasome RNA-seq read counts in two biological replicates.
(c) Quantitative comparison of the RNA species in migrasomes vs. cytosol. In general, 1 μg total RNA can be isolated from purified migrasomes containing 20 μg protein, and can then be used for RNA-sequencing or RT-PCR. KAPA Library Quantification Kits were used for library quantification. Six pre-diluted DNA standards and appropriately diluted NGS libraries were amplified using platform-specific qPCR primers that target adapter sequences. For each RNA, the read count (log10) from RNA-seq of cytosol is shown on the X-axis, and the fold change (log2) of the RNA-seq read count in migrasomes compared to cytosol is shown on the Y-axis. Significantly differentially expressed genes are marked in cyan (p-value < 0.05), and the others are marked in grey (p-value ≥ 0.05).
(d) Enrichment of Gene Ontology (GO) functional annotations in the RNA species that are enriched in migrasomes compared to the cytosol. Each circle, representing a GO term, is color-coded according to the P-value (-log10) of the GO enrichment. The size of each circle represents the proportion of gene transcripts annotated to the respective GO term in the migrasome-enriched RNA.

Figure S7

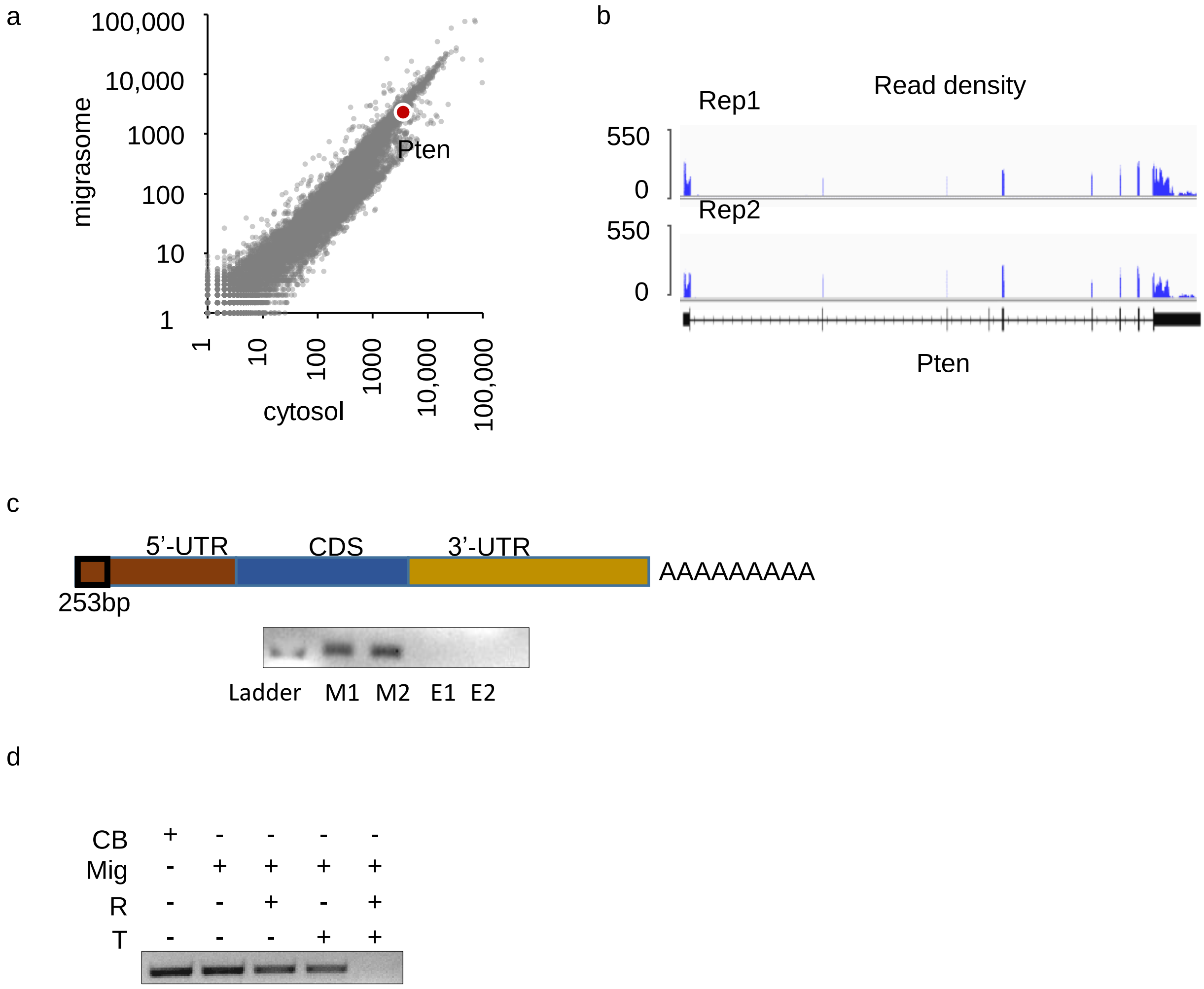

a
100,000
10,000
1000
100
10
1
migrasome
Pten
1
10
100
1000
10,000
100,000
cytosol
b
Read density
Rep1
550
0
Rep2
550
0
Pten
c
5'-UTR
CDS
3'-UTR
AAAAAAAAA
253bp
Ladder
M1
M2
E1
E2
d
CB + - - - -
Mig - + + + +
R - - + - +
T - - - + +

Supplementary information, Fig. S7. Migrasomes contain abundant and full-length *Pten* mRNA.

(a) RNA-seq read counts in L929 cytosol (X-axis) and migrasomes (Y-axis). *Pten* is highlighted on the dot plot.

(b) Density of L929 migrasome RNA-seq reads, from 2 biological replicates, aligned to the *Pten* gene body on Integrative Genomics Viewer (IGV).

(c) RT-PCR of the 5'UTR of *Pten* mRNA. Total RNA (1 μg) extracted from migrasomes and small EVs was reverse transcribed into cDNA using oligo-dT, and a pair of primers targeting the 5'-UTR of the *Pten* mRNA was used for PCR. The PCR products were run on a gel. Two independent repeat experiments were performed. M1, migrasome repeat 1; M2, migrasome repeat 2; E1, small EVs repeat 1; E2, small EVs repeat 2.

(d) Total RNA was extracted from cell bodies (CB), purified migrasomes (Mig), purified migrasomes treated with 10 μg/ml RNase A (R), purified migrasomes treated with 0.1% Triton X-100 (T), or purified migrasomes treated with 10 μg/ml RNase plus 0.1% Triton X-100. The same amount of migrasomes was used for each treatment. For cell bodies, the same amount of isolated RNA was used as for untreated migrasomes (Mig). RT-PCR was then carried out to detect *Pten* mRNA in the different samples.

Figure S8

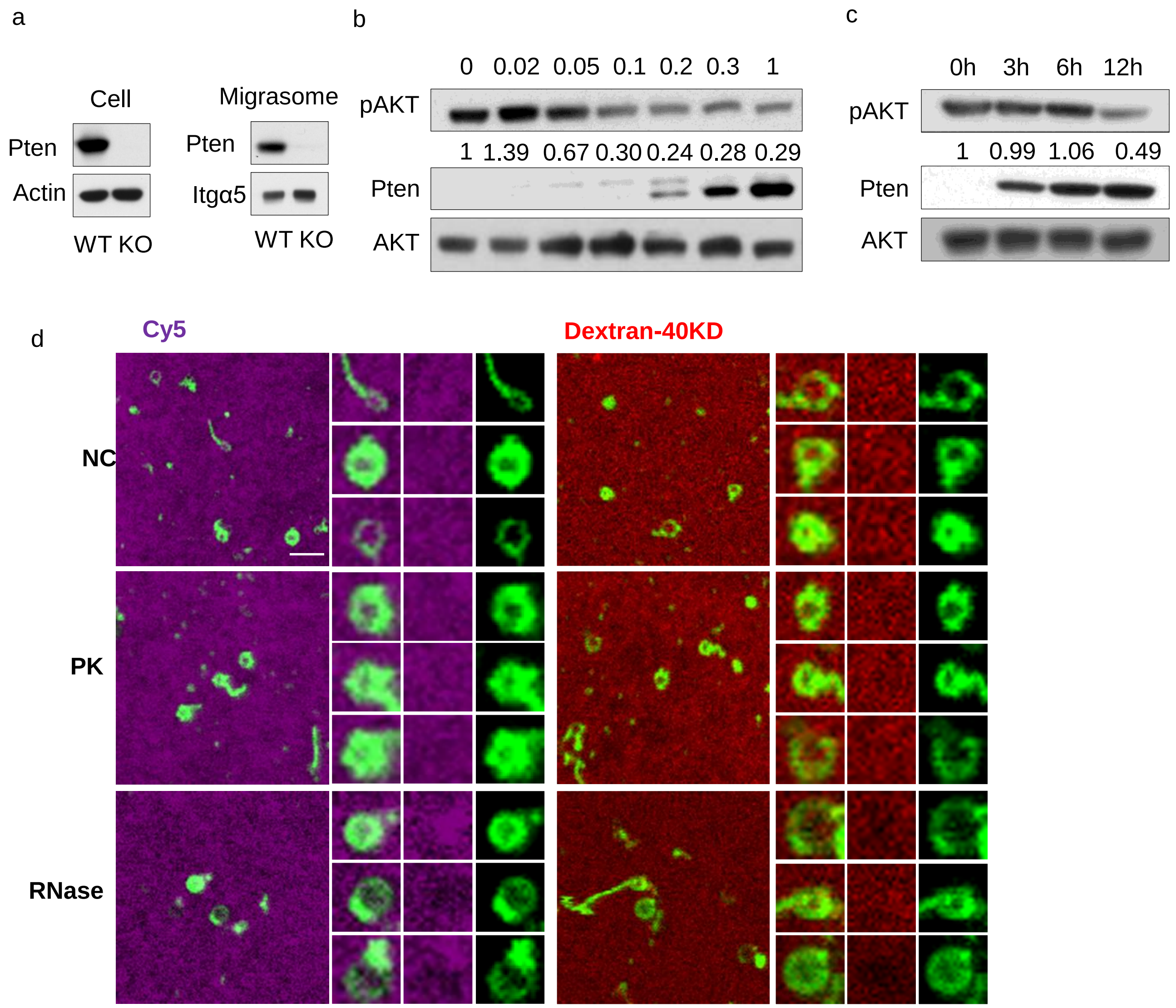
a
Cell
Pten
Actin
WT KO
Migrasome
Pten
Itgα5
WT KO
b
0 0.02 0.05 0.1 0.2 0.3 1
pAKT
1 1.39 0.67 0.30 0.24 0.28 0.29
Pten
AKT
c
0h 3h 6h 12h
pAKT
1 0.99 1.06 0.49
Pten
AKT
d
Cy5
Dextran-40KD
NC
PK
RNase

Supplementary information, Fig. S8. Dose- and time-dependent effect of migrasome in recipient cells.

(a) Left panel: WT cells and *Pten* KO cells were analyzed by western blot with antibodies against Pten and Actin. Right panel: migrasomes from WT cells and *Pten* KO cells were analyzed by western blot with antibodies against Pten and Integrin α5.

(b) Recipient cells were seeded into 96-well plates and allowed to reach 30% confluence 12 hours before treatment with migrasomes. Purified migrasomes (10 μg) were diluted as indicated and incubated with MDA-MB-468 cells for 12 h. Protein levels were assessed by western blotting.

(c) Recipient cells were seeded into 96-well plates and allowed to reach 30% confluence 12 hours before treatment with migrasomes. Purified migrasomes (8 μg) were incubated with MDA-MB-468 cells for 3 h, 6 h, and 12 h. Protein levels were assessed by western blotting.

(d) Migrasomes from L929 Tspan4-GFP cells were purified, then left untreated (NC), or treated with 50 μg/ml Proteinase K for 30 min, or treated with 10 μg/ml RNase for 30 min. The migrasomes were supplemented with 10 μg/ml Cy5 or 25 μg/ml 40 kD Dextran, and then observed by confocal microscopy. Scale bar, 2 μm.

Figure S9

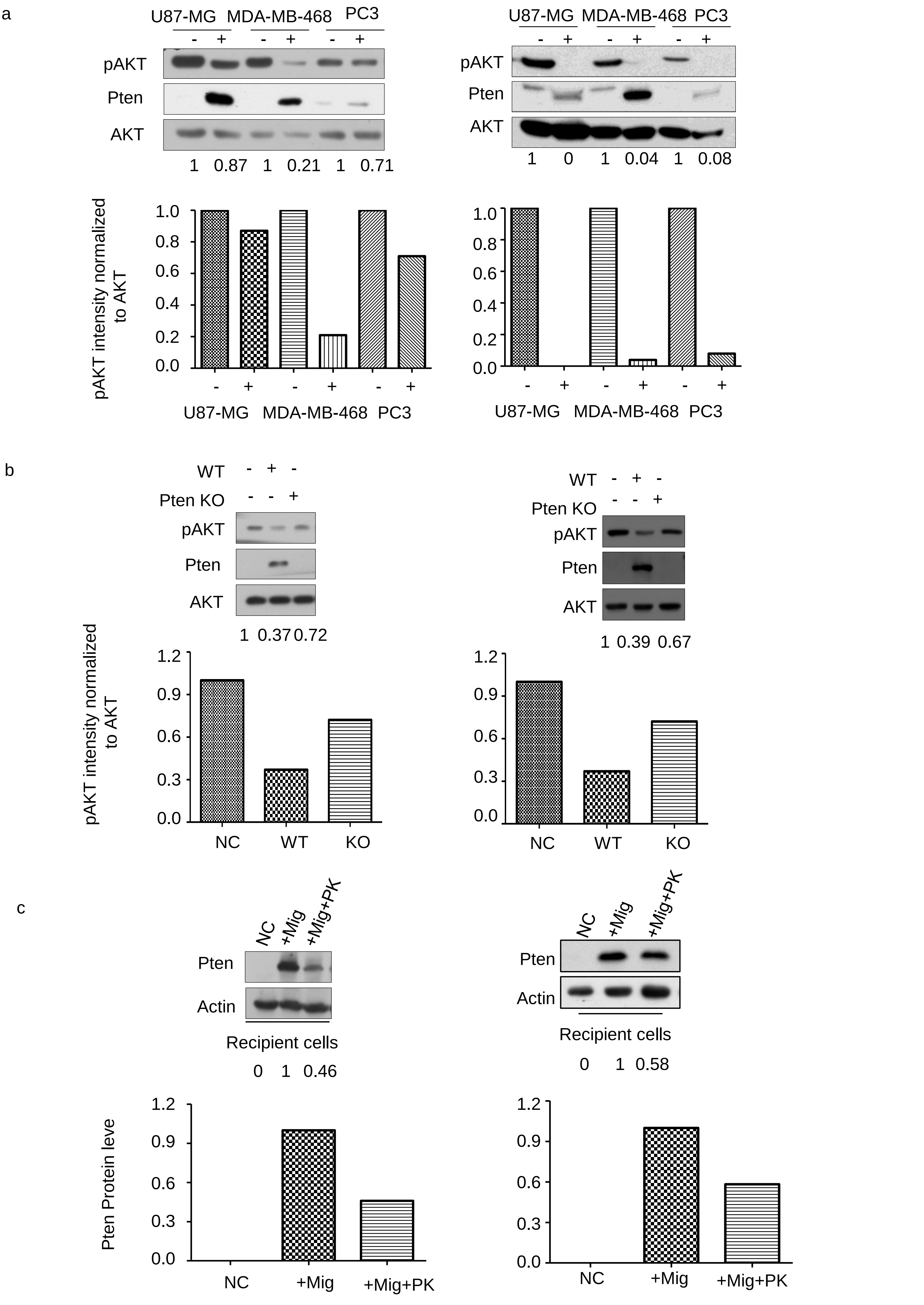
a
U87-MG
MDA-MB-468
PC3
- + - + - +
pAKT
Pten
AKT
1 0.87 1 0.21 1 0.71
1 0 1 0.04 1 0.08
pAKT intensity normalized to AKT
1.0
0.8
0.6
0.4
0.2
0.0
b
WT - + -
Pten KO - - +
1 0.37 0.72
1 0.39 0.67
1.2
0.9
0.6
0.3
0.0
NC
WT
KO
c
NC
+Mig
+Mig+PK
Pten
Actin
Recipient cells
0 1 0.46
0 1 0.58
Pten Protein leve

d

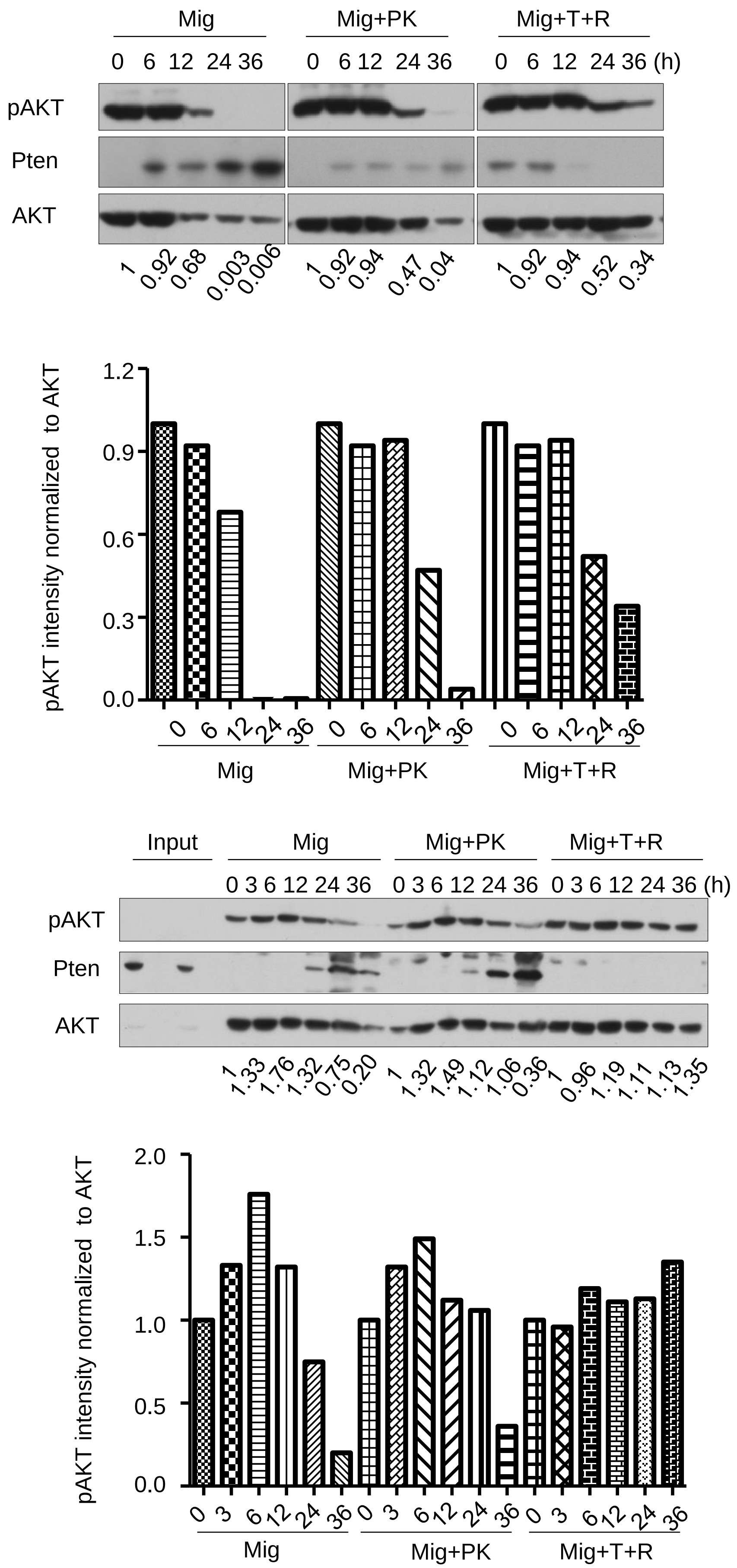

Mig
Mig+PK
Mig+T+R
0 6 12 24 36
0 6 12 24 36
0 6 12 24 36 (h)
pAKT
Pten
AKT
1 0.92 0.68 0.003 0.006
1 0.92 0.94 0.47 0.04
1 0.92 0.94 0.52 0.34
pAKT intensity normalized to AKT
1.2
0.9
0.6
0.3
0.0
0 6 12 24 36
0 6 12 24 36
0 6 12 24 36
Mig
Mig+PK
Mig+T+R
Input
Mig
Mig+PK
Mig+T+R
0 3 6 12 24 36
0 3 6 12 24 36
0 3 6 12 24 36 (h)
pAKT
Pten
AKT
1 1.33 1.76 1.32 0.75 0.20
1 1.32 1.49 1.12 1.06 0.36
1 0.96 1.19 1.11 1.13 1.35
pAKT intensity normalized to AKT
2.0
1.5
1.0
0.5
0.0
0 3 6 12 24 36
0 3 6 12 24 36
0 3 6 12 24 36
Mig
Mig+PK
Mig+T+R

e

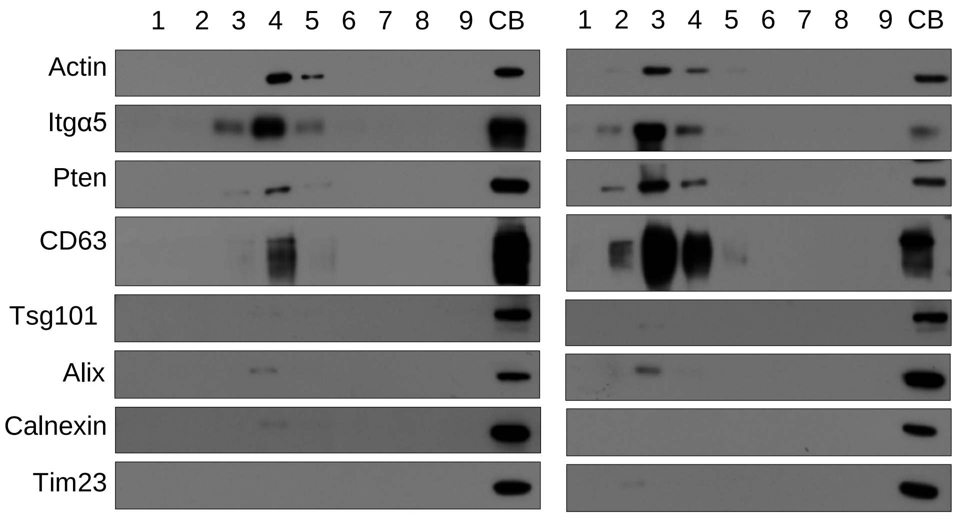

	1	2	3	4	5	6	7	8	9	CB
Actin	-0.01	0.01	0.04	0.68	0.26	0.04	0.04	0.05	0.05	1.00
Itgα5	0.10	0.33	2.30	1.49	0.46	0.05	0.00	-0.01	0.00	1.00
Pten	-0.02	0.08	0.79	0.43	0.08	0.00	-0.01	-0.02	-0.02	1.00
CD63	-0.02	0.09	0.63	0.74	0.11	0.01	0.02	0.02	0.03	1.00
Tsg101	0.00	0.00	0.01	0.02	0.00	0.00	0.00	0.00	0.00	1.00
Alix	-0.01	0.00	0.05	0.01	0.00	0.01	-0.01	0.00	0.00	1.00
Calnexin	0.00	0.01	0.03	0.06	0.02	0.02	0.01	0.00	0.01	1.00
Tim23	-0.01	-0.01	0.01	0.01	0.02	0.01	0.01	0.00	-0.01	1.00

f

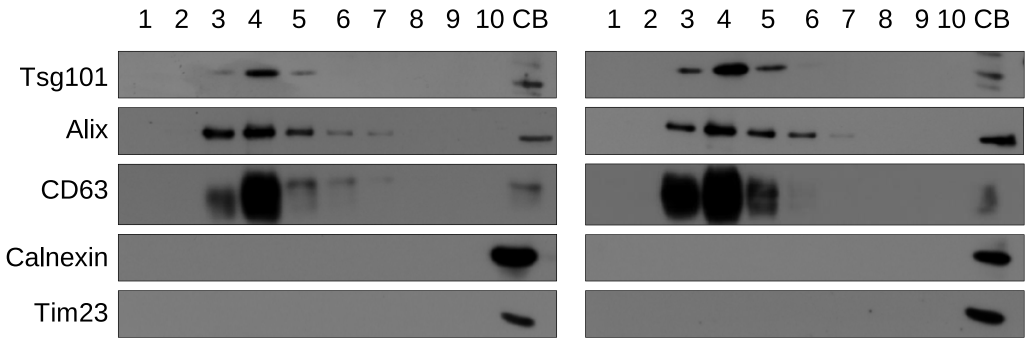

	1	2	3	4	5	6	7	8	9	10	CB
Tsg101	-0.02	0.04	0.63	2.70	0.60	0.12	0.06	-0.01	-0.01	0.00	1.00
Alix	0.00	0.00	1.34	1.94	0.99	0.43	0.18	0.10	0.09	0.09	1.00
CD63	0.06	0.16	4.08	8.96	2.96	0.68	0.27	0.14	0.11	0.08	1.00
Calnexin	0.00	0.00	0.01	0.01	0.01	0.00	0.00	0.00	0.00	-0.01	1.00
Tim23	0.00	0.02	0.05	0.05	0.06	0.06	0.07	0.08	0.08	0.08	1.00

g

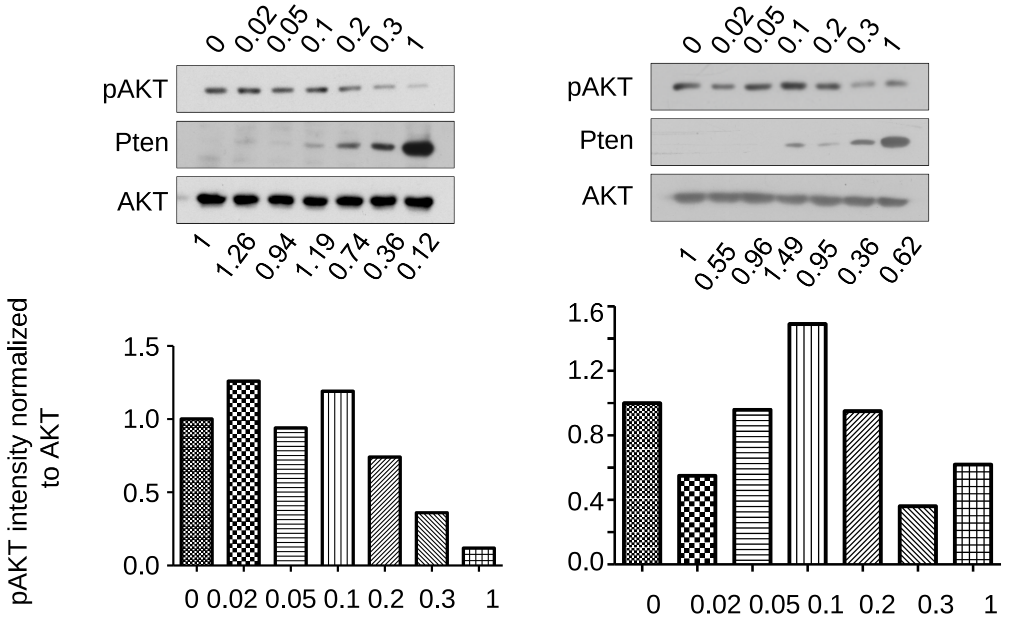

0 0.02 0.05 0.1 0.2 0.3 1
pAKT
Pten
AKT
1 1.26 0.94 1.19 0.74 0.36 0.12
0 0.02 0.05 0.1 0.2 0.3 1
pAKT
Pten
AKT
1 0.55 0.96 1.49 0.95 0.36 0.62
pAKT intensity normalized to AKT
1.5
1.0
0.5
0.0
0 0.02 0.05 0.1 0.2 0.3 1
1.6
1.2
0.8
0.4
0.0
0 0.02 0.05 0.1 0.2 0.3 1

h

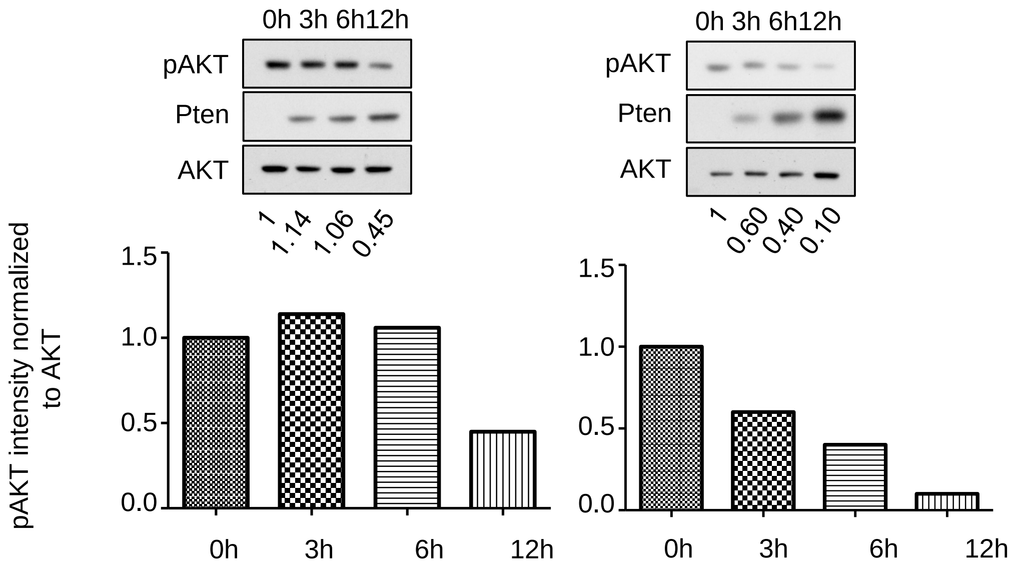

0h 3h 6h 12h
pAKT
Pten
AKT
1 1.14 1.06 0.45
0h 3h 6h 12h
pAKT
Pten
AKT
1 0.60 0.40 0.10
pAKT intensity normalized to AKT
1.5
1.0
0.5
0.0
0h 3h 6h 12h
1.5
1.0
0.5
0.0
0h 3h 6h 12h

Supplementary information, Fig. S9. Independent repeats for Figs 1i, j, l, m, S2c, d, S8b, c.

(a) Independent repeat experiments for Fig. 1i. Recipient cells were seeded into 96-well plates and allowed to reach 30% confluence 12 hours before treatment with migrasomes. Purified migrasomes (8 μg) were incubated with U87-MG, MDA-MB-468, and PC3 cells for 24 h and the recipient cells were washed twice with PBS. Protein levels were assessed by western blotting and measured by image J. The level of pAKT was normalized to AKT, and plotted as a bar graph (shown below the western blot figure).

(b) Independent repeat experiments for Fig. 1j. Recipient cells were seeded into 96-well plates and allowed to reach 30% confluence 12 hours before treatment with migrasomes. Migrasomes (8 μg) were purified from WT or *Pten* KO L929 cells and incubated with MDA-MB-468 cells for 24 h. The recipient cells were then washed twice with PBS. pAKT and Pten were analyzed by western blotting and protein levels were measured by image J. The level of pAKT was normalized to AKT and plotted as a bar graph (below the western blot figure).

(c) Independent repeat experiments for Fig. 1l. Purified migrasomes (10 μg) treated with or without 50 μg/ml Proteinase K for 30 min were washed twice with PBS and incubated with MDA-MB-468 cells for 24 h. Cellular Pten levels were assessed by western blotting. Actin was used as the loading control. NC, negative control (untreated cells). Protein levels were measured by image J. The level of Pten was normalized to Actin and plotted as a bar graph (below the western blot figure).

(d) Independent repeat experiments for Fig. 1m. Recipient cells were seeded into 96-well plates and allowed to reach 30% confluence 12 hours before treatment with migrasomes. Purified migrasomes (20 μg) were treated with 50 μg/ml Proteinase K (Mig+PK) or 10 μg/ml RNase plus 0.1% Triton X-100 (Mig+T+R), washed twice with PBS and incubated with MDA-MB-468 cells for 3 h, 6 h, 12 h, 24 h, and 36 h. Protein levels were assessed by western blotting and measured by image J. The level of pAKT was normalized to AKT and plotted as a bar graph (shown below the western blot figure).

(e) Independent repeat experiments for Fig. S2c. Crude migrasomes were centrifuged in an OptiPrep density gradient. The different fractions (1-9) were analyzed by western blotting to determine the distribution of the cytosol marker Actin, the migrasome enriched protein Itga5, the ER marker Calnexin, the small EV markers CD63, Alix and Tsg101, and the mitochondrion marker Tim23. CB, cell bodies. The level of each marker in the fraction was measured by Image J and normalized to the level in cell bodies. The data are presented as a heatmap, which shows the average levels of each marker from all 3 repeats.

(f) Independent repeat experiments for Fig. S2d. Small EVs collected from the cell culture medium were centrifuged in a sucrose density gradient. The distribution of the small EVs markers CD63, Alix and Tsg101, the ER marker Calnexin, and the mitochondrion marker Tim23 in the fractions was monitored by western blotting. The level of each marker in the fractions was measured by Image J and normalized to the level in the cell body. The results are presented as a heat map, which shows the average relative levels of each marker from all 3 repeats.

(g) Independent repeat experiments for Fig. S8b. Purified migrasomes (10 μg) were diluted as indicated and incubated with MDA-MB-468 cells for 12 h. Protein levels were assessed by western blotting and measured by image J. The level of pAKT was normalized to AKT and plotted as a bar graph, which is shown below the western blot figure.

(h) Independent repeat experiments for Fig. S8c. Recipient cells were seeded into 96-well plates and allowed to reach 30% confluence 12 hours before treatment with migrasomes. Purified migrasomes (8 μg) were incubated with MDA-MB-468 cells for 3 h, 6 h, and 12 h. Protein levels were assessed by western blotting and measured by image J. The level of pAKT was normalized to AKT and plotted as a bar graph (shown below the western blot figure).

MATERIALS AND METHODS

Reagent and antibodies. SYTO 14 (S7576) was from Thermo Fisher Scientific. Fibronectin (PHE0023) was from Life Technologies. GLPG0187 was from MedChemexpress (HY-100506). Anti-Pten (9188S), Anti-pAKT-S473 (4060P), Anti-AKT (9272S), Anti-Itga5 (#4705), Anti-Annexin v (8555S) were from Cell Signaling Technology. Anti-β-Actin (DD0208) was from Zen Bioscience. Anti-CD63 (ab217345), Anti-Calnexin (ab22595), Anti-PIGK (ab201693), Anti-Annexin A1 (ab214486) were from Abcam. Anti-CPQ (HPA023235-100μl) was from Sigma. Anti-Tsg101 (GTX70255) was from Gene Tex. Anti-Tim23 (611222), Anti-flotillin1 (610820) were from BD Transduction Laboratories. The antibodies described above were used at 1:1000 for western blotting. Blebbistatin (T6038) was from TargetMol.

Cell culture. L929 cells and MDA-MB-468 cells were cultured in DMEM (Life Technologies) supplemented with 10% FBS (5% CO_2). U87-MG cells were cultured in MEM (ThermoFisher) supplemented with 10% FBS and NEAA. PC3 cells were cultured in RPMI-1640 (HyClone) supplemented with 10% FBS.

Live-cell imaging. Cells were plated in 35 mm glass-bottom dishes coated with fibronectin (10 μM/ml) the night before imaging and maintained at 37 °C with 5% CO_2. Images were acquired using Dragonfly spinning disk microscopy.

SYTO 14 labeling and WGA staining. L929 fibroblast cells were cultured in 10 μg/ml

fibronectin-coated dishes overnight and fixed by 4% paraformaldehyde for 15 min. Fixed cells were washed with PBS twice and incubated with 2.5 μM SYTO 14 or 1 μg/ml WGA (a dye which stains migrasomes) for 10 min. Images were acquired using Nikon A1 or Olympus Fv1200 confocal microscopes with a 60× objective. 637 nm laser and Alexa Fluo 647 channel were used for WGA647 imaging; 488 nm laser and Alexa Fluo 488 channel were used for SYTO 14 imaging.

Annotation of migrasomes to cells. For each individual cell, the total number of migrasomes and the number of SYTO 14-positive migrasomes were counted. The percentage of SYTO 14-positive migrasomes was calculated as: (number of SYTO 14-positive migrasomes / total number of migrasomes) ×100.

RNase and DNase treatment. Cells were prepared and fixed as described above. After fixation, cells were permeabilized with 0.1% saponin for 10 min and then incubated with 2.5 μM SYTO 14. Images were captured with confocal microscopy and set as 0 min. Then cells were treated with RNase A (CWBIO, CW0600S) at a final concentration of 10 μg/ml or RNaseFree DNase (Promega, M6101) at a final concentration of 30 u/ml. PBS was used as the buffer for RNase treatment, and PBS supplemented with 10× DNase reaction buffer was used as the buffer for DNase treatment (this buffer contains 1 mM Ca^{2+} at the final concentration), according to the manual. Images were captured every 20 min for 1 h.

Migrasome purification. Cells seeded on fibronectin-coated dishes were cultured overnight, then the culture medium was disposed of and the cells were washed with PBS. After trypsinization, the samples were centrifuged at 1000×g for 10 min to remove cell bodies, and then 4000×g for 20 min to remove cell debris, and then 18000×g for 40 min to collect crude migrasomes. The pellet, containing crude migrasomes, was resuspended in 800 µl sample buffer (400 µl 10% OptiPrep + 400 µl extraction buffer) and fractionated at 150000×g using an MLS50 rotor (Optima MAX-XP) for 4 h in an OptiPrep density gradient. The gradient was: 5% (500 µl), 10% (sample, 800 µl), 15% (500 µl), 20% (500 µl), 25% (500 µl), 30% (500 µl), 35% (500 µl), 40% (500 µl), 50% (500 µl). Fractions were prepared for negative staining, western blotting analysis, BCA assay, or RNA extraction.

Purification of Small EVs. Cells were cultured in DMEM supplemented with 10% small EVs-free FBS for 36 h. The medium was collected and centrifuged at 1000×g for 10 min, and then 4000×g for 20 min, and then 100000×g in a 45Ti rotor (Optima XPN-80) for 60 min. The pellet, containing crude small EVs, was resuspended in 100 µl PBS and then mixed with 400 µl 2M sucrose and fractionated at 150000×g using an MLS50 rotor (Optima MAX-XP) for 4 h in a sucrose density gradient. The gradient was: 0.25 M (400 µl), 0.5 M (400 µl), 0.75 M (400 µl), 1 M (400 µl), 1.25 M (400 µl), 1.5 M (400 µl), 1.75 M (400 µl), 2 M (sample, 500 µl), 2.25 M (400 µl), 2.5 M (400 µl). Fractions were prepared for negative staining and western blotting analysis.

Microvesicle purification. Cells seeded on fibronectin-coated dishes were cultured overnight in DMEM supplemented with 10% FBS filtered through a 0.22-μm filter. The medium was collected and centrifuged at 1000×g for 10 min to remove cell bodies, and then 4000×g for 20 min to remove cell debris, and 18000×g for 40 min to collect microvesicles. The pellet was washed by PBS twice, then resuspended in 100 μl PBS, and prepared for negative staining and western blotting analysis.

Negative staining and TEM imaging. Pellets containing purified migrasomes or small EVs were resuspended in 50-100 μl PBS, then a 5 μl sample of each was mixed with the same volume of 2.5% glutaraldehyde (PB buffer, pH 7.4), and fixed for 30 min at room temperature. The sample was spread onto glow-discharged Formvar-coated copper mesh grids (Electron Microscopy Sciences, Hatfield) for about 5 min, then washed with water. The sample was then stained with 1% uranyl acetate for 2 min. Excess staining solution was blotted off with filter paper and the copper mesh grids were washed with water. After drying, grids were imaged at 10-100 kV using a transmission electron microscope H-7650.

BCA assay for migrasome quantitation. Purified migrasomes were lysed in 2% SDS buffer, and subjected to Pierce™ BCA Protein Assay (ThermoFisher, 23224, 23228) according to the manufacturer's instructions. In detail, Albumin BSA was diluted in 2% SDS buffer to prepare standards of 0 μg, 5 μg, 10 μg, 25 μg, 50 μg, 75 μg, 100 μg, 200 μg. BCA Reagent A and Reagent B were mixed in a 50:1 ratio to prepare the working

reagent. Then 80 µl working reagent and 10 µl sample were mixed in a 96-well plate, and incubated at 37 °C for 30 min. The absorbance was measured by microplate reader at 562 nm. Then a standard curve was generated from the BSA standards and used to determine the migrasome concentration.

RNA extraction. Total RNA was extracted from migrasomes and small EVs by TRIzol Reagent (Invitrogen, 15596026) according to the manufacturer's instructions. In detail, the samples were suspended and homogenized in 500 µl of TRIzol reagent. 100 µl of chloroform was added and the samples were vortexed vigorously for 15 seconds and incubated on ice for 5 min. The samples were centrifuged at 12,000×g for 15 minutes at 4 °C. The upper aqueous phase (250 µl) was carefully transferred into a fresh tube and the RNA was precipitated with 250 µl isopropyl alcohol supplemented with 1 µg RNase-free glycogen at -20 °C for 2 hours. The samples were centrifuged at 12,000×g for 15 minutes at 4 °C and the supernatant was removed completely. The RNA pellet was washed twice with 75% ethanol, air-dried for 5 min, then dissolved in RNase-free water.

RT-PCR. Reverse transcription reactions were performed using a RevertAid First Strand cDNA Synthesis Kit (Thermo scientific, K1622) according to the manufacturer's instructions. 1 µg of total RNA was reverse transcribed using oligo dT or random primers as the primer. Primers for quantitative PCR analysis of Pten cDNA were as follows: forward CAGTCTCTGCAACCATCCA, reverse

ATAAATATAGGTCAAGTCTAAGTCG. 20 PCR cycles were performed. Primers for the 5'-end of the *Pten* mRNA were: forward CTTTTTTAGTGAGGAAACAATT, reverse TTCCTAAAGCAAGTTATTCATCC.

Generation of L929 cells with stable knockout of *Pten*. The CRISPR/Cas9 system was a gift from Dr Wensheng Wei's lab (Peking University). The sgRNA-binding sequence for CRISPR/Cas9-mediated mutation of the *Pten* gene was 5'-ATTTCCTGCAGAAAGACTTGA-3'. Cas9, gRNA and puromycin plasmid were co-transfected into L929 cells. Cells were cultured in medium containing 6 μg/ml puromycin for about 10 days, after which cell clones were picked and transferred into 96-well plates for amplification and analysis by western blotting.

Migrasome incubation. Recipient cells were seeded into 96-well plates and allowed to reach 30% confluence 12 hours before treatment with migrasomes. Purified migrasomes were washed twice with PBS and incubated with recipient cells for 24 h. Then the recipient cells were washed twice with PBS. Protein levels were assessed by western blotting.

Analysis of cell proliferation by EdU incorporation. MDA-MB-468 cells were incubated with migrasomes (8 μg) purified from WT or *Pten* KO L929 cells for 18 h and then treated with EdU (10 μM final concentration) for 2 h. EdU labeling and staining of cells were performed using EdU Proliferation Assay Kits (iFluor 488,

ab219801) and captured by High Content Opera Phenix microscopy. The total cell number was derived by counting the DAPI-stained nuclei, and the number of EdU-positive cells was counted from the same images.

Immunofluorescence staining. L929 fibroblast cells were cultured in 10 μg/ml fibronectin-coated dishes overnight and fixed by 4% paraformaldehyde for 15 min at room temperature, and permeabilized with 0.05% saponin for 30 min. Fixed cells were blocked with 10% FBS in PBS for 30 min, stained with 10 μg/ml antibody in blocking buffer for 1 hr, and washed with PBS. Cells were then stained with secondary antibody in blocking buffer for 1 hr and washed with PBS three times.

smFISH RNA in situ hybridization. Single-molecule fluorescence in situ hybridization was performed as previously described [1] with modifications. In brief, cells cultured in fibronectin-coated glass-bottom dishes were fixed in 1×PBS solution containing 4% (wt/vol) paraformaldehyde for 30 min at room temperature, washed with 1×PBS, and permeabilized at 4 °C in 70% (vol/vol) ethanol overnight. On the following day, cells were washed three times with wash buffer [2×SSC, 10% (vol/vol) formamide] and then incubated in hybridization buffer [10% (wt/vol) dextran sulfate, 2×SSC, 10% (vol/vol) formamide] containing a pool of singly-TAMRA-labeled oligonucleotides complementary to different regions of the *Pten* coding sequence (total probe concentration = 100 nM, Supplementary information, Table S2) for 24 h at 37 °C in a humidified chamber. Slides were washed with wash buffer followed by 2×SSC to

remove any unbound probes and incubated in 1×PBS prior to imaging.

Length distribution analysis of RNA. Electropherograms of total RNA isolated from the cytosol, migrasome and small EVs fractions of mouse L929 cells are generated by an Agilent 2100 Bioanalyzer. Size distributions of the RNAs are shown on the electropherogram traces by red lines, and the percentages of long (>200 nt) and short (<200 nt) RNA species were calculated according to the grey value of the bands on the RNA gel.

Total RNA sequencing library preparation. Total RNA was extracted from the migrasomes and the cytosolic fraction from the same cells. The sequencing libraries were assessed with a BioAnalyzer and quantified using a KAPA SYBR FAST Universal qPCR Kit (Kapa Biosystems, KK4601) prior to and after pooling for sequencing. Library insert sizes were typically about 30 bp. The pooled libraries were sequenced on the Illumina HiSeq 2500 platform with a single-end sequencing strategy for 50 cycles.

Processing of RNA-seq data. Low-quality reads with Phred quality scores lower than 20 (>50% of bases) were removed using the fastx quality filter (http://hannonlab.cshl.edu/fastx_toolkit/). The reads originating from rRNAs were identified and discarded by aligning the reads to mouse rRNA sequences (5S, 5.8S, 18S, and 28S) using Bowtie (version 1.1.2) with no mismatch allowed. The remaining reads were then mapped to the mouse genome and spliced transcripts using STAR with the

following parameters: --outFilterType BySJout --outFilterMismatchNmax 2 --outSAMtype BAM --quantMode TranscriptomeSAM --outFilterMultimapNmax 1 --outFilterMatchNmin 16. To control the noise from multiple alignments, reads mapped to multiple genomic positions were discarded.

Statistical Analysis. Statistical analysis was performed in Graphpad Prism. Experimental groups were compared using two-tailed t-tests (Fig. 1o; Supplementary information, Fig. S3c and S4b, e) or two-way ANOVA test (Fig. 1d). All data were obtained from independent experiments. SYTO 14 intensity in migrasomes or cell bodies, and the protein levels on western blots, were measured by Image J software. 3D reconstructions were generated by IMARIS software.

Data availability. The RNA sequencing data generated in this study have been deposited in the Gene Expression Omnibus (GEO) with the accession code GSE130753. All the data analysis results are provided in the supplementary files.

REFERENCE

1 Raj, A.et al. *Nature methods* **5**, 877-879 (2008).
