# Supplementary figures and images for: Lateral transfer of mRNA and protein by migrasomes modifies the recipient cells
Source: Cell Res. 2020 Sep 29;31(2):237–40. doi: 10.1038/s41422-020-00415-3 (PMC8026638; doi:10.1038/s41422-020-00415-3)

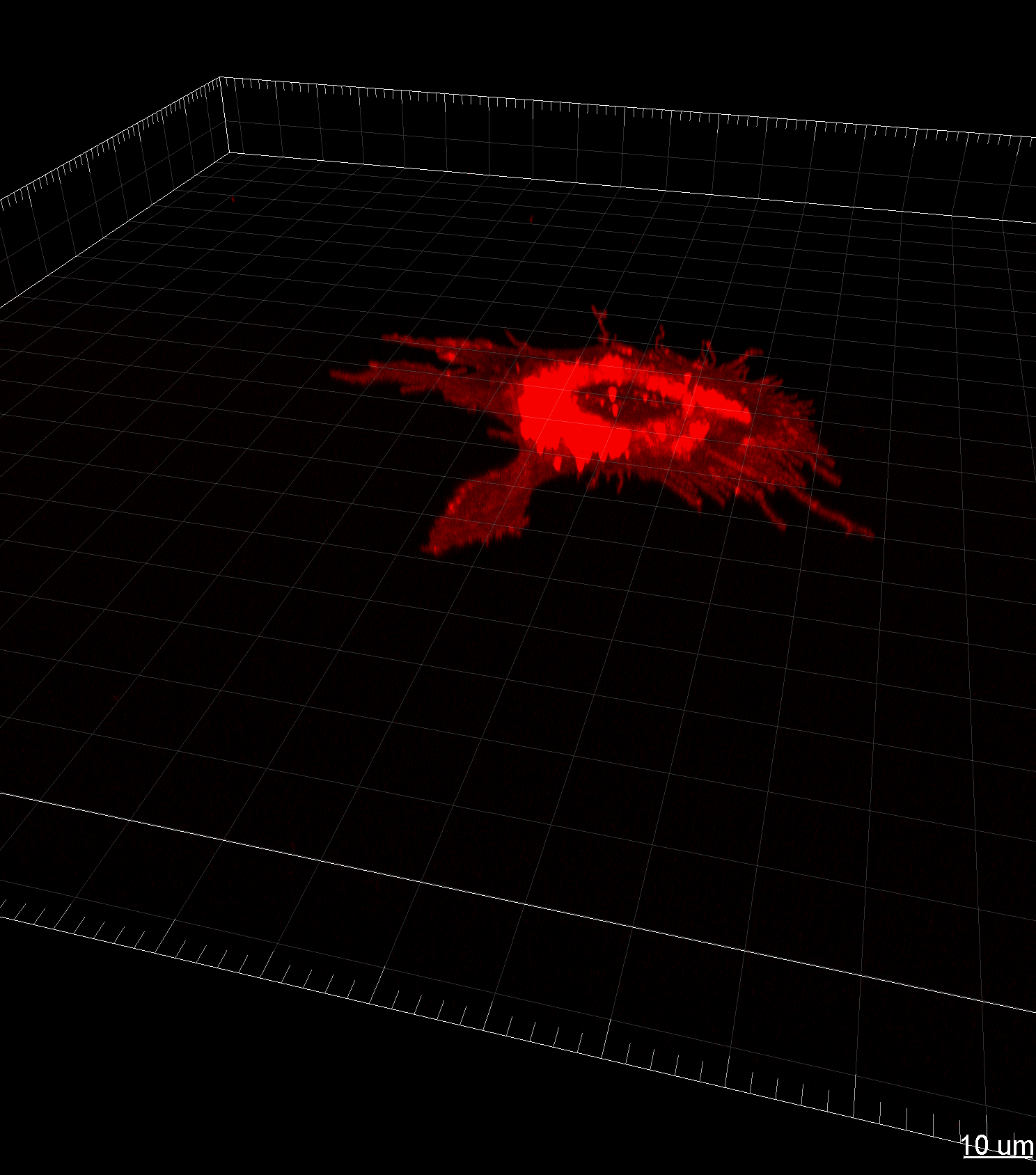

Supplement: Supplementary file 4 — Supplementary information, movie S1 [file 41422_2020_415_MOESM4_ESM.gif]
