# Supplementary material for: Lateral transfer of mRNA and protein by migrasomes modifies the recipient cells
Source: Cell Res. 2020 Sep 29;31(2):237–40. doi: 10.1038/s41422-020-00415-3 (PMC8026638; doi:10.1038/s41422-020-00415-3)
Supplement: Supplementary file 5 — Supplementary information, movie S1 legend [file 41422_2020_415_MOESM5_ESM.docx]

Supplementary information, movie S1

A representative 4-dimensional movie of an L929 cell expressing the migrasome marker Tspan4-mCherry. Tspan4-mCherry-expessing L929 cells were cultured in 10 μg/ml fibronectin-coated dishes for 4 h and then time-lapse images were acquired using Dragonfly spinning disk microscope. Images were captured every 10 min for 12 h. Scale bar, 10 μm. Related to Fig. S3a.
